# Specificity of Mimotope-Induced Anti-High Molecular Weight-Melanoma Associated Antigen (HMW-MAA) Antibodies Does Not Ensure Biological Activity

**DOI:** 10.1371/journal.pone.0019383

**Published:** 2011-05-06

**Authors:** Julia Latzka, Sonja Gaier, Gerlinde Hofstetter, Nina Balazs, Ursula Smole, Soldano Ferrone, Otto Scheiner, Heimo Breiteneder, Hubert Pehamberger, Stefan Wagner

**Affiliations:** 1 Department of Pathophysiology and Allergy Research, Center for Pathophysiology, Infectiology and Immunology, Medical University of Vienna, Vienna, Austria; 2 Department of Surgery, of Immunology and of Pathology, University of Pittsburgh Cancer Institute, Pittsburgh, Pennsylvania, United States of America; 3 Division of General Dermatology, Department of Dermatology, Medical University of Vienna, Vienna, Austria; Wistar Institute Program, United States of America

## Abstract

Vaccines based on peptide mimics (mimotopes) of conformational tumor antigen epitopes have been investigated for a variety of human tumors including breast cancer, tumors expressing the carcinoembryonic antigen, B cell lymphoma, neuroblastoma, and melanoma. In our previous work, we designed a vaccine based on a mimotope of the high molecular weight-melanoma associated antigen (HMW-MAA) that elicited HMW-MAA-specific antibodies (Abs) with anti-tumor activity *in vitro* and *in vivo*. In this study, we aimed to identify mimotopes of additional distinct HMW-MAA epitopes, since they could be used to construct a polymimotope melanoma vaccine. For this purpose, random peptide phage libraries were screened with the anti-HMW-MAA monoclonal antibodies (mAbs) VT80.12 and VF1-TP43 yielding one peptide ligand for each mAb. Both peptides inhibited the binding of the corresponding mAb to the HMW-MAA. Furthermore, when coupled to the carrier protein keyhole limpet hemocyanin (KLH), both HMW-MAA mimotopes elicited peptide-specific Abs in rabbits or BALB/c mice, but only the mimotope isolated with the mAb VT80.12 elicited HMW-MAA-specific Abs and only in mice. However, the latter Abs had no detectable effect on HMW-MAA expressing human melanoma cells *in vitro*. These results describe limitations related to the phage display technique and emphasize the need to characterize the functional properties of the mAb utilized to isolate mimotopes of the corresponding epitopes.

## Introduction

For at least two decades, the high molecular weight-melanoma associated antigen (HMW-MAA) has been the focus of several studies to implement effective immunotherapy for melanoma. Originally identified with murine monoclonal antibodies (mAbs) on the surface of human melanoma cell lines, HMW-MAA has been found to be expressed in at least 80% of primary and metastatic melanomas and more recently, in several other tumors as well as on cancer stem cells [1], [2]. However, HMW-MAA expression is not restricted to transformed cells, as it has been found on various cells of normal tissues including melanocytes, hair follicles and basal cells of the epidermis as well as endothelial cells, chondrocytes and pericytes [1]. Its expression on both activated and resting pericytes in tumor vessels has suggested that HMW-MAA is critical to tumor angiogenesis [2]. Furthermore, HMW-MAA is known to contribute to the malignant phenotype of melanoma by activating several signaling cascades (e.g. Rho GTPases, p130^cas^, FAK) that are involved in adhesion, migration and invasion of melanoma cells [3].

The possibility that HMW-MAA represents a useful target to implement immunotherapy of melanoma has been initially suggested by the association between development of HMW-MAA-specific antibodies (Abs) and statistically significant survival prolongation in patients with advanced melanoma immunized with a mouse anti-idiotypic mAb which mimics a HMW-MAA epitope [4], [5]. This possibility has been further supported by the results of several preclinical studies which have targeted HMW-MAA with Ab- and T cell-based immunotherapeutic strategies. Recently, Maciag *et al.* generated a *Listeria monocytogenes* (*Lm*)-based vaccine against HMW-MAA [6]. Immunization of C57BL/6 mice bearing B16F10-HMW-MAA melanomas induced HMW-MAA-specific CD8^+^ and CD4^+^ T cells that were equally required for tumor inhibition, as *in vivo* depletion of each of these cells resulted in uncontrolled tumor growth [6]. Noteworthy, in this study for the first time a syngeneic melanoma mouse model for HMW-MAA was used and novel information on the contribution of T cells to the therapeutic efficacy of HMW-MAA-specific immunity was provided.

In contrast, we have emphasized the induction of HMW-MAA-specific humoral immunity by using mimotopes as immunogens. Mimotopes are small peptides that mimic conformational B cell defined-epitopes of antigens and can be selected by screening random peptide phage libraries with a mAb of interest. As mimotopes do not necessarily share the identical amino acid sequence with the original antigen, they represent useful antigen surrogates to overcome immunotolerance to self-antigens [7]–[10]. Regarding melanoma, this is of particular interest as most melanoma associated antigens including the HMW-MAA are self-antigens [1], [11].

In previous studies, we have identified a linear HMW-MAA mimotope (225D9.2+) which mimics the epitope recognized by the HMW-MAA-specific mAb 225.28S. Immunization with this mimotope coupled to tetanus toxoid induced HMW-MAA-specific Abs in rabbits that inhibited melanoma cell proliferation *in vitro* [12]. Passive administration of these Abs in a xenogeneic melanoma SCID mouse model inhibited tumor growth up to 40% and 62% in a therapeutic and prophylactic setting, respectively [13].

As epitope loss is commonly found in melanoma cells [14] and studies have shown that induction of antibodies against multiple epitopes of a breast cancer tumor antigen increased tumor growth inhibition [15], [16], efficacy of a melanoma vaccine might be improved by vaccination with several peptides. In this regard, we report the selection of mimotopes of additional HMW-MAA epitopes which are distinct from that defined by the mAb 225.28S and discuss limitations related to mimotope vaccines regarding their immunogenicity and antigenicity.

## Materials and Methods

### Ethics statement

Mice were treated according to European Union Rules of Animal Care and all experiments were approved by the Animal Experimentation Committee of the Medical University of Vienna and the Austrian Ministry of Science (permission 66.009/152-II/10b/2009).

### Monoclonal anti-HMW-MAA Abs

The mAbs VT80.12, VF1-TP43, and TP61.5 were developed and characterized as described elsewhere [17]–[19].

### Biotinylation of Abs (mAbs, rabbit IgG)

NHS-LC-Biotin (Pierce, Rockford, IL, USA) was diluted in dimethylformamide at a concentration of 40 mg/ml. Five microliters of this solution was added to 1 mg/ml mAb or rabbit IgG in PBS and incubated for 45 min at room temperature (RT). Excess NHS-LC-Biotin was removed by dialysis against PBS.

### Cell lines

The human melanoma cell line 518A2 [20] which expresses high levels of HMW-MAA and M14 [21], a human melanoma cell line with no detectable expression of HMW-MAA, were maintained in RPMI 1640 medium (Lonza, Verviers, Belgium) supplemented with 10% (v/v) FCS and 1% (v/v) antibiotic-antimycotic mix (both from Gibco, Paisley, UK). Both cell lines were cultured in a humidified atmosphere containing 5% CO_2_ and 95% ambient air at 37°C.

### Microsomal preparations

Microsomal preparations were performed using ∼5×10^7^ cells according to the protocol described elsewhere [22]. Protein concentration was determined using a bicinchoninic acid (BCA) protein assay (Pierce).

### Phage display, affinity selection and sequence analysis

Peptide ligands for the mAb VT80.12 were selected from a pVIII-15mer phage display peptide library [23]. Therefore, the mAb VT80.12 was immobilized and incubated with ∼10^10^ phages. Phages displaying peptides that bound to the mAb VT80.12 were eluted and amplified in *E. coli* TG1.

Peptide ligands for the mAb VF1-TP43 were selected using the Ph.D.-12™ Phage Display Peptide Library, a pIII-12mer library, purchased from New England Biolabs (Ipswich, MA, USA). Biopanning was performed following the manufacturer's instructions.

After three rounds of selection, single phage clones that bound to the respective mAb and not to the isotype-matched control mAb in phage ELISA were subsequently subjected for DNA sequencing. Obtained sequences were not deposited in GenBank.

### Synthesis of peptides

The peptides GRQYYEGRKPDYRAAC (15/3/6) and NYQDLQRTHFKSGPGPGC (43.12p3) were synthesized using F-moc strategy by piCHEM (Graz, Austria). The purity of the peptides was ∼95%, as assessed by HPLC.

### ELISA inhibition assay

MaxiSorp immunoplates (Nunc, Rosklide, Denmark) were coated overnight (o/n) at 4°C with mAb T61.5 (4 µg/ml in 50 mM Na-carbonate buffer, pH 9.6). Biotinylated mAb (10 ng) was incubated o/n at 4°C with increasing concentrations (10, 50, 100, and 500 µg/ml) of synthetic peptides in TBST (0.5% (v/v) Tween-20) containing 1% (w/v) BSA. After blocking with TBST/3% (w/v) milk powder, plates were incubated for 3 h at RT with microsomal preparations (100 µg/ml in TBST/1% BSA) to catch HMW-MAA. After washing, the mAb preincubated with peptides was added to the plate and incubation was continued for an additional hour at RT. Bound biotinylated mAb was detected using alkaline phosphatase (AP)-conjugated streptavidin (GE Healthcare, Little Chalfont, UK), followed by the addition of p-nitrophenylphosphate (Sigma). Absorbance was measured at 405 nm. Percentage of inhibition was calculated as follows: 100−(OD (inhibited)/OD (uninhibited)×100).

### Conjugation of peptides

Peptides were coupled to the carrier protein keyhole limpet hemocyanin (KLH; Sigma, St. Louis, MO, USA) or bovine serum albumin (BSA; Pierce) using the heterobifunctional crosslinker reagent m-maleimidobenzoyl-N-hydroxysuccinimide (MBS; Pierce) as described previously [12]. Conjugation of the peptides to the carrier proteins was verified in a dot blot assay as follows. Peptide conjugates were dotted onto nitrocellulose (NC) membrane (Whatman, Dassel, Germany). After blocking with PBST (0.5% Tween-20) containing 3% milk powder, NC strips were incubated with biotinylated mAb, followed by AP-conjugated streptavidin (GE Healthcare). Color development was done with 5-bromo-4-chloro-3-indolyl phosphate/nitroblue tetrazolium.

### Immunizations

Female New Zealand white rabbits (2 groups, n = 1; 13–17 weeks old) were immunized s.c. at the Charles River Laboratories (Kisslegg, Germany) on day 1, 29, and 43 with 200 µg peptide-KLH conjugate or KLH alone adsorbed to CFA (for priming) and to IFA (for boosting). Serum samples were harvested before treatment (preimmune serum), on day 11 and 39. Rabbits were sacrificed on day 57.

Female BALB/c mice (4 groups, n = 3; 6–8 weeks old; Charles River Laboratories, Sulzfeld, Germany) were immunized i.p. on day 1, 15, and 29 each with 15 µg of the peptide-KLH conjugates or KLH alone adsorbed to aluminum hydroxide (alum; Serva, Heidelberg, Germany) in a total volume of 150 µl PBS solution. Sham-treated mice received PBS buffered alum only. Blood samples were taken on day 0 (preimmune serum), 22 and 41, and mice were sacrificed on day 48.

### Purification of rabbit or mouse IgG Abs

Total IgG were purified from sera of the immunized rabbits according to the protocol described elsewhere [12].

Mouse IgG were purified from serum samples taken after the third immunization or after sacrifice of mice. Therefore, sera from the three mice of each group were pooled and diluted with an equal volume of binding buffer (20 mM Na-phosphate, pH 7.0). The HiTrap Protein G HP column (GE Healthcare) was equilibrated with binding buffer and the sample applied. Bound IgG were eluted with 100 mM glycine-HCl, pH 2.7 and neutralized by the addition of 1 M Tris-HCl, pH 9.0.

Purification of rabbit and mouse IgG was monitored using nonreducing 8% SDS-PAGE. Protein concentration was determined by a BCA protein assay (Pierce).

### Peptide-specific Ab response

MaxiSorp immunoplates (Nunc) were coated o/n at 4°C with 10 µg/ml peptide-BSA conjugates or KLH or BSA in 50 mM Na-carbonate buffer, pH 9.6. Nonspecific binding sites were blocked with TBST/3% milk powder. Purified rabbit IgG (0.05–10 µg/ml in TBST/0.5% BSA) or mouse sera (diluted 1∶1000 in TBST/0.5% BSA) were added to antigen-coated plates and incubated for 2 h at RT. After washing, bound Abs were detected using AP-conjugated swine anti-rabbit IgG (diluted 1∶1000 in TBST/0.5% BSA; Dako, Glostrup, Denmark) or AP-conjugated rabbit anti-mouse IgG+IgM (diluted 1∶5000 in TBST/0.5% BSA; Jackson ImmunoResearch, West Grove, PA, USA). Color development was performed as described for the ELISA inhibition assay.

### HMW-MAA-specific Ab response

#### ELISA protocol

For the detection of the HMW-MAA-specific Ab response, HMW-MAA was purified from microsomal preparations as described for the ELISA inhibition assay. After blocking and catching, plates were incubated for 2 h at RT with increasing concentrations (12.5, 50, and 200 µg/ml) of purified rabbit IgG diluted in TBST/1% BSA. Bound IgG was detected as described for the peptide-specific Ab response.

#### FACS protocol

Flow cytometric analysis of melanoma cells stained with sera from immunized mice or purified IgG from immunized rabbits was performed as previously described [24]. Briefly, 5×10^5^ cells were incubated for 1 h on ice with a 1∶10 dilution of pooled mouse sera for each group or 100 µg purified rabbit IgG in 100 µl PBS/1% BSA. MAb VT80.12 (1 µg) served as positive control staining of HMW-MAA. Cells were then washed twice with PBS/0.5% BSA and incubated for an additional 30 min on ice with FITC-labeled goat anti-mouse IgG or FITC-labeled goat anti-rabbit IgG (both diluted 1∶2000 in PBS/1% BSA; AbD Serotec, Düsseldorf, Germany). After washing, cells were resuspended in PBS/0.5% BSA and 20,000 gated events were analyzed by flow cytometry on a BD FACScanto using BD FACSDiva software (BD Biosciences, Franklin Lakes, NJ, USA). Histogram overlays were done applying FlowJo software (Tree Star, Ashland, OR, USA).

#### Immunohistochemical (IHC) staining

IHC staining of solid 518A2 tumors established in C.B.17 SCID/SCID mice was performed as described by Wagner *et al.* [13]. Briefly, tumor sections were incubated with either blocking buffer (negative control), biotinylated mAb VT80.12 (30 µg/ml; positive control) or biotinylated IgG (100 µg/ml) purified from rabbits either immunized with 15/3/6-KLH conjugate or KLH. After washing with TBST, slides were incubated with StreptABComplex/HRP (Dako) for 30 min. Specific Ab binding was visualized by a DAB chromogen solution (Dako) following hematoxylin counterstaining. Stained slides were viewed by an Olympus Vanox AHBT3 microscope and photographed with a Zeiss AxioCam MRc5 camera.

### Inhibition of tumor cell growth in vitro

Tumor cells were seeded in 96-well tissue culture plates (Costar; Corning, NY, USA) at 1500 cells/well. Cells were allowed to adhere o/n at 37°C. IgG purified from mice either immunized with 15/3/6-KLH conjugate or KLH were added at increasing concentrations (0.01, 0.1, and 1 mg/ml) and incubation was continued for additional 72 h at 37°C. Cells were pulsed with 0.5 µCi of [methyl-^3^H]thymidine/well (GE Healthcare) for another 6 h at 37°C and then harvested. Incorporated [^3^H]thymidine was measured by a Wallac MicroBeta TriLux 1450 counter (PerkinElmer, Waltham, MA, USA). Percentage of inhibition of proliferation was calculated by comparing cpm values of treated cells with those of untreated cells, which were set at 100%.

## Results

### Identification of peptide ligands

**Figure 1:**
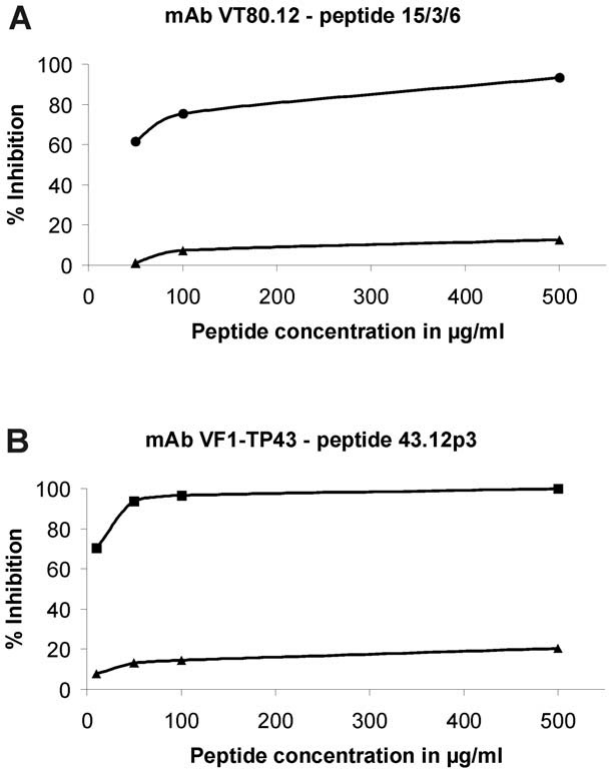
Inhibition of mAb binding to HMW-MAA by synthetic peptides. Microtiter plates were coated with mAb TP61.5 and incubated with microsomal preparation of 518A2 melanoma cells to catch HMW-MAA. Biotinylated mAbs were preincubated with increasing concentrations of synthetic peptides, followed by incubation with HMW-MAA. (**A**) Biotinylated mAb VT80.12 was preincubated with peptide 15/3/6 (•) or an irrelevant control peptide (▴). (**B**) Biotinylated mAb VF1-TP43 was preincubated with peptide 43.12p3 (▪) as well as the control peptide (▴). Binding of biotinylated mAbs was measured using an AP-conjugated streptavidin. Percentage of inhibition was calculated as follows: 100−(OD (inhibited)/OD (uninhibited)×100).

Biopanning of the mAb VT80.12 was performed with a linear pVIII-15mer phage display peptide library. After three rounds of selection, the total number of phages that bound to the mAb VT80.12 was increased from 1×10^5^ CFU/ml in the 1^st^ round to 1×10^9^ CFU/ml in the 3^rd^ round. Twenty individual phage clones of each the 2^nd^ and 3^rd^ round were tested for their ligand specificity in phage ELISA. Among these, eighteen (2^nd^ round) and nineteen (3^rd^ round) phage clones specifically bound to the mAb VT80.12. DNA sequencing of these 37 phage clones yielded one peptide sequence GRQYYEGRKPDYRAA (“15/3/6”). The peptide 15/3/6 was chemically synthesized with an additional C-terminal cysteine residue for conjugation purposes. In ELISA inhibition experiments, this peptide inhibited the binding of the mAb VT80.12 to the HMW-MAA up to 93% (Figure 1A).

Biopanning of the mAb VF1-TP43 was performed with a linear pIII-12mer phage display peptide library. Three rounds of selection yielded an increase of phage titer from 1×10^5^ pfu/ml (1^st^ round) to 3×10^9^ pfu/ml (3^rd^ round). Among 30 tested phage clones, 20 were specifically recognized by the mAb VF1-TP43 in phage ELISA. DNA sequencing yielded one peptide sequence NYQDLQRTHFKS (“43.12p3”). The peptide 43.12p3 was synthesized with a GPGPG-linker to improve presentation on the surface of the carrier protein and an additional C-terminal cysteine residue. In subsequent inhibition experiments, this peptide inhibited the binding of the mAb VF1-TP43 to the HMW-MAA up to 100% (Figure 1B).

### Peptide-specific Ab response in rabbits

**Figure 2:**
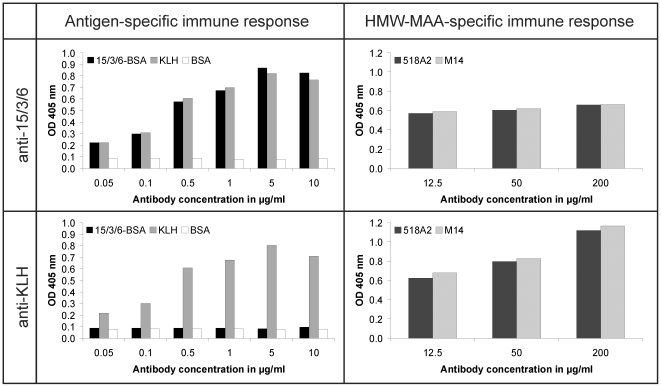
Antigen- and HMW-MAA-specific immune response of immunized rabbits. **Left panel:** Microtiter plates were coated with 15/3/6-BSA conjugate, KLH or BSA and incubated with increasing concentrations of purified rabbit IgG from immunizations with 15/3/6-KLH conjugate or KLH to detect peptide- and KLH-specific Abs. **Right panel:** Microtiter plates were coated with mAb TP61.5 and incubated with microsomal preparations of the melanoma cell lines 518A2 or M14. Purified rabbit IgG were analyzed by administration of increasing Ab concentrations.

The peptide 15/3/6 was coupled to KLH or BSA as carrier protein. Conjugation to the carrier protein was confirmed in a dot blot assay (data not shown). New Zealand white rabbits were immunized with the 15/3/6-KLH conjugate or the carrier protein KLH alone. After three immunizations, IgG were purified using a HiTrap protein A HP column. Twenty milliliters of serum yielded ∼20 mg of IgG Abs. The purity was greater than 95% as confirmed by SDS-PAGE (data not shown). Peptide-specific Abs were determined by ELISA after incubation of coated peptide-BSA conjugate, BSA or KLH with purified IgG Abs. BSA-conjugates were used to ensure detection of peptide-specific IgG. Both, 15/3/6- and KLH-specific Abs were already detectable at a concentration of 0.05 µg/ml and their levels increased in a dose-dependent manner (Figure 2). Abs purified from the KLH-immunized rabbit did not bind to the peptide. No Abs directed against BSA could be determined.

### HMW-MAA-specific Ab response in rabbits

HMW-MAA specificity of purified rabbit IgG was determined in ELISA by testing their binding ability to HMW-MAA purified from microsomal preparations of 518A2 cells. 15/3/6-KLH conjugate induced Abs bound to the HMW-MAA, but also when the HMW-MAA catching mAb was incubated with microsomal preparations of M14 cells (HMW-MAA^neg^), binding of these Abs was detected (Figure 2). The same results were observed for the KLH-induced Abs (Figure 2). Even when the HMW-MAA catching mAb was directly incubated with the purified rabbit IgG and subsequently detected with AP-conjugated swine anti-rabbit IgG, high background levels were measured (data not shown).

**Figure 3:**
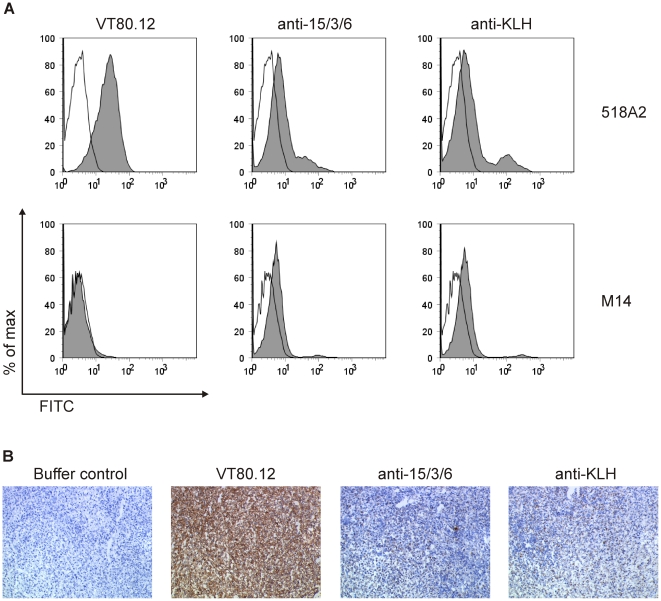
HMW-MAA-specific Ab response determined by FACS and IHC. (**A**) The human melanoma cell lines 518A2 and M14 were each incubated with 1 µg mAb VT80.12 (as positive control to detect HMW-MAA) or 100 µg purified rabbit IgG from immunizations with 15/3/6-KLH conjugate or KLH. Bound IgG were detected with FITC-labeled goat anti-mouse IgG or FITC-labeled goat anti-rabbit IgG. Histogram overlays show unstained (white) vs. stained cells (grey). (**B**) IHC on 518A2 tumor tissues of C.B.17 SCID/SCID mice [13]. HMW-MAA staining was done with PBS buffer (negative control), mAb VT80.12 (positive control), and biotinylated IgG purified from rabbits immunized with 15/3/6-KLH conjugate (anti-15/3/6) or KLH (anti-KLH). Bound Abs were detected with StrepAB/HRP and visualized by DAB-chromogen solution and subsequent hematoxylin counterstaining.

In a second approach, HMW-MAA specificity of rabbit IgG was investigated by FACS analysis. The HMW-MAA positive human melanoma cell line 518A2 (HMW-MAA^pos^) or the HMW-MAA negative human melanoma cell line M14 (HMW-MAA^neg^) were incubated with each 100 µg purified rabbit IgG and detected with a FITC-labeled goat anti-rabbit IgG. As expected, the mAb VT80.12 specifically bound to the HMW-MAA^pos^, but not to the HMW-MAA^neg^ cells. However, HMW-MAA specificity of the 15/3/6-KLH conjugate induced Abs could not be observed, as Abs induced by KLH showed a similar staining pattern to HMW-MAA^pos^ as well as to HMW-MAA^neg^ cells (Figure 3A).

In a third approach, we used solid 518A2 tumors established in C.B.17 SCID/SCID mice [13] to detect HMW-MAA-reactive rabbit Abs by IHC. Whereas staining of melanoma cells was observed with the biotinylated mAb VT80.12, none was observed with either an isotype-matched control mAb (data not shown) or PBS buffer. Also in this setup, we detected a background staining of anti-KLH Abs on 518A2 tumor tissue that was comparable to the staining of the anti-15/3/6-KLH Abs (Figure 3B).

### Peptide-specific Ab response in BALB/c mice

**Figure 4:**
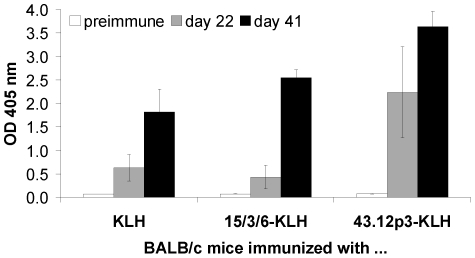
Antigen-specific immune response of immunized BALB/c mice. Serum samples from day 0 (preimmune serum; white bars), day 22 (grey bars), and day 41 (black bars) were tested in ELISA. The mean value of the individual responses of each group is shown and standard deviations are indicated by error bars. Peptide specificity of sera from mice either immunized with 15/3/6- or 43.12p3-KLH conjugate was assessed using the respective peptide-BSA conjugates. Sera from KLH immunized mice were tested for specificity to KLH.

The peptides 15/3/6 or 43.12p3 were coupled each to KLH or BSA. Female BALB/c mice were immunized with 15 µg of the peptide-KLH conjugates, KLH or were sham-treated with PBS buffered alum. Serum samples of each group were tested for the presence of peptide-specific Abs in ELISA. Peptide specificity was confirmed for Abs induced by the 15/3/6- and the 43.12p3-KLH conjugate, as they bound to the corresponding peptide-BSA conjugates (Figure 4). As expected, KLH-specific Abs were detected in the KLH-group (Figure 4) as well as in the groups immunized with the peptide-KLH conjugates (data not shown). No peptide- (15/3/6 or 43.12p3), KLH- or BSA-specific Abs were detected in the sera of sham-treated mice (data not shown).

### HMW-MAA-specific Ab response in BALB/c mice

**Figure 5:**
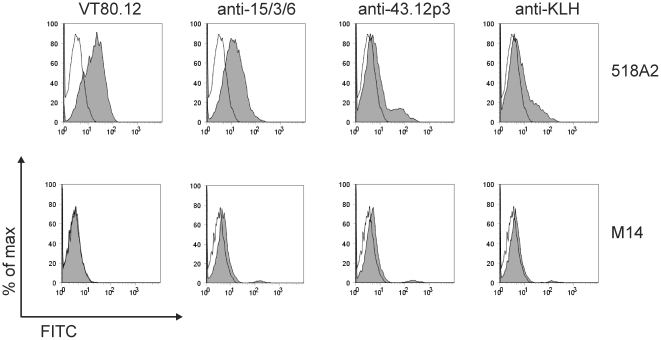
HMW-MAA-specific immune response of immunized BALB/c mice. Human melanoma cell lines 518A2 and M14 were either incubated with 1 µg mAb VT80.12 (as positive control) or a 1∶10 dilution of pooled mouse sera of each group. Bound IgG were detected with FITC-labeled goat anti-mouse IgG. Histogram overlays show unstained (white) and stained cells (grey).

Preimmune serum samples or serum samples after the third immunization of individual mice were pooled for each group. Serum pools were diluted 1∶10 and incubated with HMW-MAA^pos^ or HMW-MAA^neg^ cells. Bound IgG were detected with a FITC-labeled goat anti-mouse IgG by FACS analysis. No staining was detected of HMW-MAA^pos^ or HMW-MAA^neg^ melanoma cells by mouse preimmune serum pools (data not shown). The 15/3/6-KLH conjugate induced HMW-MAA-specific Abs, whereas the 43.12p3-KLH conjugate did not (Figure 5). KLH-induced Abs showed negligible background staining on HMW-MAA^pos^ as well as HMW-MAA^neg^ cells.

### Inhibition of tumor cell growth

**Figure 6:**
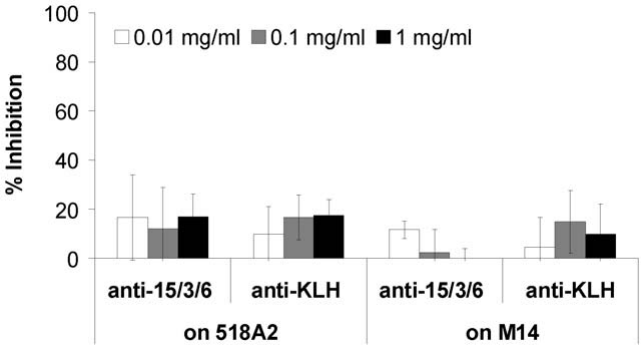
Inhibition of melanoma cell proliferation *in vitro*. [^3^H]thymidine proliferation assay demonstrating effects of purified mouse IgG on HMW-MAA expressing melanoma cells. 518A2 or M14 cells were incubated with increasing concentrations of purified IgG from mice immunized with 15/3/6-KLH conjugate or KLH for 72 h and pulsed with [^3^H]thymidine. Data are presented as percentage of inhibition of proliferation compared to untreated cells. Percentage of inhibition was calculated as follows: 100−(cpm (treated)/cpm (untreated)×100). Values represent the mean of three independent experiments.

The effect of anti-15/3/6-KLH Abs on tumor growth *in vitro* was determined by a [^3^H]thymidine proliferation assay. HMW-MAA^pos^ or HMW-MAA^neg^ cells were incubated with increasing concentrations of purified mouse IgG. Upon treatment with anti-15/3/6 Abs, proliferation of HMW-MAA^pos^ cells was inhibited to a maximum of ∼17% (Figure 6). Almost identical results were obtained, when either HMW-MAA^pos^ cells or HMW-MAA^neg^ cells were treated with anti-KLH Abs.

## Discussion

In this study we aimed to select two mimotopes defined by the HMW-MAA-specific mAbs VT80.12 and VF1-TP43 that should serve together with our previously described 225D9.2+ mimotope [12], [13] as components of a polymimotope vaccine against melanoma.

We therefore screened two linear phage display peptide libraries (pVIII-15mer with mAb VT80.12, pIII-12mer with mAb VF1-TP43) yielding one peptide ligand for each mAb. Both peptides (15/3/6: ligand to VT80.12; 43.12p3: ligand to VF1-TP43) inhibited binding of the respective mAb to the HMW-MAA up to 100%, thereby proofing epitope mimicry and their definition as mimotopes. Upon immunization of either rabbits or BALB/c mice, each mimotope coupled to the carrier protein KLH demonstrated immunogenicity by inducing mimotope-specific Abs. However, only the 15/3/6-KLH conjugate induced HMW-MAA-specific Abs in BALB/c mice, but these Abs failed to inhibit tumor cell proliferation *in vitro*.

Epitope mimicry to HMW-MAA was confirmed for both peptides 15/3/6 and 43.12p3 in ELISA inhibition experiments (Figure 1). An important issue of this study was the applied animal model in the context of the carrier protein used for vaccination. Initially, immunization experiments were performed in rabbits as high amounts of antibodies can be obtained from the blood for subsequent *in vitro* tests. Although mimotope-specific Abs were induced for the tested mimotope, these showed no HMW-MAA specificity in ELISA experiments (Figure 2). Instead, we observed a high background staining of KLH-specific Abs on the HMW-MAA-positive or -negative cell lysate, as well as on the HMW-MAA catching mAb. Similarly, FACS staining (Figure 3A) as well as IHC (Figure 3B) failed to confirm HMW-MAA-specific Abs. Immunizations were then repeated in BALB/c mice to assess whether the results from the rabbit immunizations were attributed to the carrier protein KLH or the applied animal model. Using this model, FACS analysis confirmed HMW-MAA specificity of mimotope-induced Abs (Figure 5), indicating that the undesired binding of rabbit anti-KLH Abs was likely attributed to the rabbit Abs and not to the carrier protein KLH. However, we cannot exclude a possible contribution of the used adjuvants (CFA/IFA in rabbits vs. alum in BALB/c mice) to the unspecific binding of rabbit anti-KLH Abs, as adjuvants are known to differentially deliver antigen and activate the immune system [25]. We therefore conclude that the choice of carrier protein has an impact on the outcome of vaccination and might depend on the used immunization model.

Although the 43.12p3-KLH conjugate induced mimotope-specific mouse Abs, these failed to cross-react with the natural antigen HMW-MAA (Figure 5), indicating that immunogenicity of mimotopes does not necessarily correlate with their specificity. The discrepancy between epitope specificity of an anti-HMW-MAA mAb and epitope specificity of HMW-MAA mimotope-induced Abs is best described by previous studies of Wagner *et al.* [12] and Hafner *et al.* [26]. Both studies deal with the identification of distinct HMW-MAA mimotopes which were defined by the same mAb (225.28S) and coupled to the same carrier protein (tetanus toxoid, TT) for subsequent immunization experiments in rabbits. Using the identical immunization protocol, a HMW-MAA-specific Ab response was only obtained upon immunization with the mimotope-TT conjugate described by Wagner *et al.* [12]. These findings clearly indicate that – despite confirmed affinity selections as well as analog immunization protocols – not every mimotope truly mimics the relevant epitope of the original antigen. On the one hand, limitations might originate during affinity selection as phage displaying peptides may bind to residues of the mAb that are not part of the paratope. Although such peptides might inhibit the binding of their defined mAb to the original epitope, these will fail to induce a specific immune response to the original antigen. In addition, B cell epitopes are known to be conformational in nature [27]. Hence, it is possible that mAbs recognize certain conformations of epitopes which are not displayed in the same structural context by the natural antigen. The above-described limitations probably outline the major pitfall of the phage display technique that complicates the selection of “true” mimotopes, solely based on their binding characteristics to mAbs. On the other hand, it is to state that immunization with mimotopes does not guarantee the induction of Abs that display the appropriate paratope to the natural antigen, emphasizing also a possible contribution of the used animal model to the outcome of mimotope vaccination. This might be of particular relevance for other investigators who apply mimotopes for vaccination strategies.

Immunization of BALB/c mice with the 15/3/6 mimotope conjugate resulted in the induction of mimotope- and HMW-MAA-specific Abs (Figure 4 and 5). However, these Abs showed no anti-tumor activity in *in vitro* proliferation assays (Figure 6). Importantly, the mAb VT80.12 itself does not inhibit tumor cell proliferation neither directly nor indirectly by mediating ADCC or CDC [28]. We therefore have to question whether anti-tumor activity *in vivo* of the mimotope defining mAb should be a prerequisite to induce antibodies with tumor inhibiting properties. As a matter of fact, certain mAbs (e.g. Rituximab [29]–[32], Cetuximab [33], [34], Trastuzumab [10], [35]), which are currently applied for cancer therapies and have successfully served as sources to generate mimotope vaccines with anti-tumor activity *in vitro*, fulfill this requirement, even though *in vivo* results for these mimotope vaccines are not yet available. As reported by Hafner *et al.*, passive administration of the anti-HMW-MAA mAb 225.28S significantly reduced tumor volume in a human melanoma xenotransplant SCID mouse model, although this mAb had no effect on human melanoma cells *in vitro* [36]. This supports the hypothesis of using mAbs with *in vivo* rather than *in vitro* anti-tumor activity for the selection of mimotopes.

To date, only one mimotope-based vaccine targeting the HMW-MAA has demonstrated anti-tumor activity *in vitro* [12] and *in vivo* [13]. The identification of additional HMW-MAA mimotopes as components of an effective polymimotope vaccine might depend on the *in vivo* anti-tumor activity of the selecting mAbs *per se* but also on the used animal model as well as on the paratope conformation of the mimotope-induced Abs. In addition, we suggest that the efficacy of every mimotope-based vaccine should be evaluated in an animal model by active immunization.
